# Association of the National Dependent Coverage Expansion With Insurance Use for Sexual and Reproductive Health Services by Female Young Adults

**DOI:** 10.1001/jamanetworkopen.2020.30214

**Published:** 2020-12-18

**Authors:** Jacqueline E. Ellison, Amresh D. Hanchate, Lewis E. Kazis, Megan B. Cole

**Affiliations:** 1 Department of Health Services, Policy, and Practice, Brown University School of Public Health, Providence, Rhode Island; 2 Department of Social Sciences and Health Policy, Wake Forest School of Medicine, Winston-Salem, North Carolina; 3 Department of Health Law, Policy, and Management, Boston University School of Public Health, Boston, Massachusetts

## Abstract

**Importance:**

Sexual and reproductive health services are a primary reason for care seeking by female young adults, but the association of the 2010 Patient Protection and Affordable Care Act Dependent Coverage Expansion (ACA-DCE) with insurance use for these services has not been studied to our knowledge. Insurer billing practices may compromise dependent confidentiality, potentially discouraging dependents from using insurance or obtaining care.

**Objective:**

To evaluate the association between implementation of ACA-DCE and insurance use for confidential sexual and reproductive health services by female young adults newly eligible for parental coverage.

**Design, Setting, and Participants:**

For this cross-sectional study, a difference-in-differences analysis of a US national sample of commercial claims from January 1, 2007, to December 31, 2009, and January 1, 2011, to December 31, 2016, captured insurance use before and after policy implementation among female young adults aged 23 to 25 years (treatment group) who were eligible for dependent coverage compared with those aged 27 to 29 years (comparison group) who were ineligible for dependent coverage. Data were analyzed from January 2019 to February 2020.

**Exposures:**

Eligibility for parental coverage under the ACA-DCE as of 2010.

**Main Outcomes and Measures:**

Probability of insurance use for contraception and Papanicolaou testing. Emergency department and well visits were included as control outcomes not sensitive to confidentiality concerns. Linear probability models adjusted for age, plan type, annual deductible, comorbidities, and state and year fixed effects, with SEs clustered at the state level.

**Results:**

The study sample included 4 690 699 individuals (7 268 372 person-years), with 2 898 275 in the treatment group (mean [SD] age, 23.7 [0.8] years) and 1 792 424 in the comparison group (mean [SD] age; 27.9 [0.8] years). Enrollees in the treatment group were less likely to have a comorbidity (77.3% vs 72.9%) and more likely to have a high deductible plan (14.6% vs 10.1%) than enrollees in the comparison group. Implementation of the ACA-DCE was associated with a −2.9 (95% CI, −3.4 to −2.4) percentage point relative reduction in insurance use for contraception and a −3.4 (95% CI, −3.9 to −3.0) percentage point relative reduction in Papanicolaou testing in the treatment vs comparison groups. Emergency department and well visits increased 0.4 (95% CI, 0.2-0.7) and 1.7 (95% CI, 1.3-2.1) percentage points, respectively.

**Conclusions and Relevance:**

The findings suggest that implementation of the ACA-DCE was associated with a reduction in insurance use for sexual and reproductive health services and an increase in emergency department and well health visits by female young adults newly eligible for parental coverage. Some young people who gained coverage under the expansion may not be using essential, confidential services.

## Key Points

**Question:**

Was implementation of the Patient Protection and Affordable Care Act (ACA) Dependent Coverage Expansion associated with changes in the use of insurance for confidential sexual and reproductive services among female young adults?

**Findings:**

In this cross-sectional study of commercial insurance claims for 4 690 699 individuals (7 268 372 person-years), ACA Dependent Coverage Expansion implementation was associated with a significant reduction in insurance use for contraceptive services and Papanicolaou testing and an increase in insurance use for emergency department and well visits among female young adults aged 23 to 25 years.

**Meaning:**

In this study, the ACA Dependent Coverage Expansion was associated with a reduction in insurance use for services sensitive to confidentiality concerns among female young adults targeted for expansion.

## Teaser

This cross-sectional study evaluates whether implementation of the Patient Protection and Affordable Care Act Dependent Coverage Expansion was associated with changes in the use of insurance for sexual and reproductive services among female young adults.

## Introduction

Approximately 5.5 million young adults have gained parental insurance coverage under the Patient Protection and Affordable Care Act Dependent Coverage Expansion (ACA-DCE), which requires employers to allow the children of policyholders to stay on their parents’ health plan through 26 years of age.^1^ Research suggests that ACA-DCE implementation was associated with increased office visits and use of certain preventive services, including blood pressure and cholesterol screening, but not contraceptive use, sexually transmitted infection testing, or Papanicolaou testing among young adults.^2,3,4,5,6^ Discrepancies in the types of preventive care used by young adults with parental coverage may be attributable to the sensitivity of sexual and reproductive health (SRH) services to confidentiality concerns, potentially obstructing service and/or insurance use for this confidential care. Although there has been discussion around the privacy implications of the ACA-DCE,^7,8,9^ no research to our knowledge has evaluated the association of policy implementation with insurance use for SRH services.

Understanding the association of the ACA-DCE with insurance use for SRH care is important given the implications of insurer billing practices on dependent confidentiality. Explanation of benefits forms sent to policyholders detail services provided and to whom they were provided, indirectly violating dependent confidentiality.^10^ Research consistently demonstrates a negative association between parental knowledge or involvement and SRH service use; 40% of sexually active female young adults indicated confidentiality concerns as the reason for not seeking STI testing, and 70% of adolescents with parents who were not already aware of their contraceptive use would stop using prescription contraception if parental notification of use were mandated.^11,12^ However, less attention has been paid to the role of privacy concerns in SRH care decision-making among young adults or the specific association of implementation of the ACA-DCE with insurance use behavior.

Female young adult have higher rates of unintended pregnancy and human papillomavirus infection than do their older counterparts and are more likely to experience cost-related barriers to care.^13,14^ Privacy concerns may lead those with parental coverage to delay, forgo, or pay out of pocket for services, perpetuating existing disparities.^15^ Thus, the objective of this study was to evaluate the association between the implementation of the ACA-DCE and insurance use for contraception services and Papanicolaou testing among commercially insured female young adults subsequently eligible for dependent coverage under the ACA-DCE.

## Methods

### Data and Sample

This cross-sectional study used data from the IBM MarketScan Commercial Claims and Encounters database from 2007 to 2009 and 2011 to 2016. This individual-level national database consists of employer-sponsored insurance claims and captured data on 11% to 20% of female young adults aged 20 to 29 years in the US during the study period. The database includes claims for more than 100 insurers and self-insured companies located in all 50 states. Data are fully deidentified, and thus this research was exempt from review by the Boston University institutional review board. This study followed the Strengthening the Reporting of Observational Studies in Epidemiology (STROBE) reporting guideline.

The treatment group included female young adults aged 23 to 25 years who were categorically eligible for dependent coverage under the ACA-DCE, and the comparison group included female young adults aged 27 to 29 years who were ineligible for coverage under the ACA-DCE based on their age. Because treatment status is contingent on enrollee birthday and start date of a parent’s insurance (and consequently ambiguous), female young adults aged 26 years were excluded (n = 2 153 425). Use of narrow age ranges addressed some of the methodologic limitations of prior literature on the DCE, which failed to take into account dynamics in the age structure of the health insurance and labor markets.^16^

The nature of the data source did not allow identification of individual-level insurance coverage transitions, and treatment status was therefore defined by age and subsequent eligibility for coverage under the ACA-DCE as opposed to coverage type. In other words, our analyses leveraged the substantial population-level, compositional shift in coverage type for those aged 23-25 years after implementation of the ACA-DCE. Enrollees in the comparison group with parental coverage (n = 3718) and all enrollees with spousal coverage (n = 3 033 773) were excluded, which allowed for more accurate identification of the target population of the ACA-DCE and more precise estimation of its association with aggregate SRH insurance use. Because pregnant patients are more likely to be connected with care, receive routine HIV testing, and do not use contraception, all services provided to enrollees with any evidence of delivery based on Healthcare Effectiveness Data and Information Set prenatal quality measures were excluded from the analysis.^17^ After excluding person-years with missing covariates (n = 351 340), the final study sample included 4 690 699 unique beneficiaries, with 7 268 372 person-years of enrollment from 2007–2009 and 2011–2016 (eFigure 1 in the Supplement). The final treatment group (age 23-25 years) included all person-years for those under parental or policyholder coverage (2 898 275 individuals representing 4 076 596 person-years), and the comparison group (age 27-29 years) included those with policyholder coverage (1 792 424 individuals representing 3 191 776 person-years).

### Outcomes

Outcomes of interest included Papanicolaou testing and contraception services as defined by the Healthcare Effectiveness Data and Information Set and Office of Population Affairs performance measure modalities and codes.^18,19^ We used *International Classification of Diseases, Ninth Revision, International Statistical Classification of Diseases and Related Health Problems, Tenth Revision*, Healthcare Common Procedure Coding System, Current Procedural Terminology, and National Drug Codes to identify service use (codes used to define all services are listed in eTables 9-11 in the Supplement). Contraceptive modalities included subdermal implant, intrauterine device, injectable, pill, patch, ring, and diaphragm.^17^ Although screening for sexually transmitted infection is an important sexual health service for young adults, we did not include this as an outcome owing to differing clinical guidelines between the treatment and comparison groups.^20^ We included emergency department (ED) and well visits (during which no concurrent SRH services were provided) because confidentiality concerns should not influence insurance use for this type of care.

### Statistical Analysis

We used a difference-in-differences (DID) approach to evaluate aggregate changes in insurance use for the target group of the ACA-DCE provision (age 23-25 years) before vs after ACA-DCE implementation compared with changes in a comparison group (age 27-29 years). The focus of this study was on the association between the ACA-DCE and SRH service use based on population-level, compositional changes in young adult’s type of insurance coverage after implementation of the ACA-DCE (ie, shifts from exclusively having policyholder coverage to having a mix of parental coverage and policyholder coverage). The preimplementation period was from January 1, 2007, to December 31, 2009, and the postimplementation period was from January 1, 2011, to December 31, 2016. We excluded 2010 as a washout period.

The validity of the DID study design is based on the assumption that there would have been no differing change in outcomes between the treatment and comparison groups had the intervention not occurred.^21^ We tested this assumption by statistically examining prepolicy biannual trends (eTable 1 in the Supplement) for similarity (ie, parallel trends).^22^

To assess the association of the ACA-DCE with insurance use for services, we used linear probability models, which can be interpreted as absolute percentage point changes in the probability of insurance use for each outcome (eMethods in the Supplement). The person-year was our unit of analysis. We estimated the DID with a binary indicator for use or nonuse of each service, with state and year fixed effects to account for potential unobserved heterogeneity and clustered SEs at the state-level. All models adjusted for time-variant covariates with the potential to influence service use, including age, plan type, whether or not enrollees were covered by a high-deductible plan, residence in a micropolitan or metropolitan statistical area, and a categorical variable indicating the number of comorbidities (0, 1, and ≥2) based on the Elixhauser Comorbidity Index.^23^

We conducted additional sensitivity tests to evaluate the robustness of results to DID assumptions. All analyses were replicated with the placebo outcomes (ED and well visits), which should not be sensitive to confidentiality concerns. We also repeated analyses with enrollees aged 23 to 25 years who had only parental coverage and those with only policyholder coverage in the postimplementation period to identify the extent to which aggregate changes were associated with coverage status. We replicated analyses excluding data from 2014 to 2016, which may have been subject to secondary effects of ACA coverage expansions (ie, Medicaid expansions and insurance exchanges) that could have differentially influenced the treatment and comparison groups. We also estimated the change in each individual year after implementation of the ACA-DCE compared with the preimplementation baseline to assess whether and to what extent insurance use for SRH care was a function of other policy changes occurring during the postimplementation period. In addition, to assess potential differences in care-seeking behavior between the treatment and comparison groups, we replicated all analyses excluding enrollees who did not use any services during the study period. Statistical significance was set at α = .05 with 2-tailed hypothesis tests, and analyses were performed from January 2019 to February 2020 using Stata, version 15 (StataCorp LLC).

## Results

### Sample Characteristics

**Figure 1.**
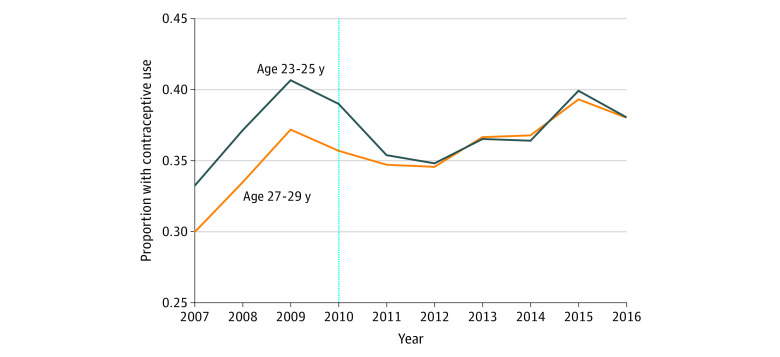
Unadjusted Trends in Insurance Use for Contraception Among Enrollees in the Treatment and Comparison Groups from 2007 to 2016 The treatment group included female young adults aged 23 to 25 years who were eligible for coverage, and the comparison group included female young adults aged 27 to 29 years who were not eligible for coverage.

**Figure 2.**
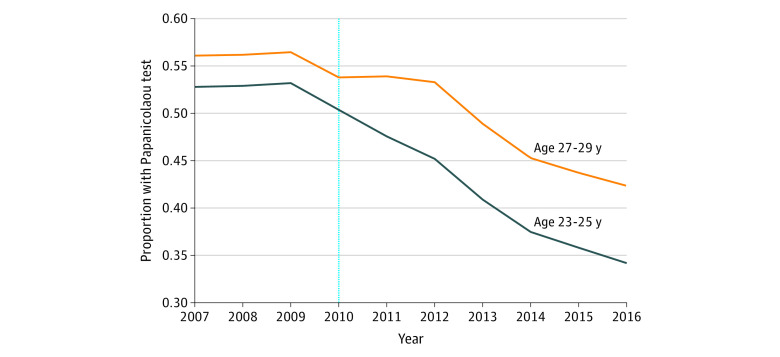
Unadjusted Trends in Insurance Use for Papanicolaou Testing Among Enrollees in the Treatment and Comparison Groups from 2007 to 2016 The treatment group included female young adults aged 23 to 25 years who were eligible for coverage, and the comparison group included female young adults aged 27 to 29 years who were not eligible for coverage.

Table 1 presents study sample characteristics stratified by the pre–ACA-DCE implementation period (2007-2009) and post–ACA-DCE implementation period (2011-2016) for enrollees in the treatment vs comparison groups; characteristics are based on an individual’s first person-year of enrollment. Those in the treatment group (age 23-25 years) were less likely to have a diagnosis of depression (7.7% vs 8.9%), more likely to have a high deductible plan (14.6% vs 10%), and less likely to have a comorbidity (22.7% vs 27.1%) than those in the comparison group (age 27-29 years). There were no other clinically meaningful differences in observed covariates between the treatment and comparison groups. The characteristics in each of these groups remained stable in the before and after implementation of the ACA-DCE with 3 exceptions. First, there was an increase in the percentage of enrollees from the Middle Atlantic and Pacific US Census regions; this is likely the result of changes in the companies included in the MarketScan data over time. Second, there was an increase in the percentage of enrollees with high deductible plans. Third, there was an increase in the proportion of enrollees in the treatment group with parental insurance coverage after ACA-DCE implementation from 17.7% before implementation to 42.5% after implementation, suggesting a compositional change in this group from during those periods (eFigure 2 in the Supplement). Although levels of insurance use were different, there were no significant preimplementation differences between the treatment and comparison groups for any of the services (Figure 1, Figure 2, and eFigures 3 and 4 in the Supplement).

**Table 1.** Characteristics of Enrollees by Treatment Status^a^

Characteristic	Treatment group (n = 2 898 275)			Control group (n = 1 792 424)		
	Total	Pre-DCE (n = 729 662)	Post-DCE (n = 2 168 613)	Total	Pre-DCE (n = 681 165)	Post-DCE (n = 1 111 259)
Age, mean (SD)	23.7 (0.8)	23.8 (0.8)	23.6 (0.7)	27.9 (0.8)	27.9 (0.8)	27.9 (0.8)
Residence in MSA						
Non-MSA	356 488 (12.3)	94 856 (13.0)	260 234 (12.0)	198 959 (11.1)	84 464 (12.4)	116 682 (10.5)
MSA	2 541 787 (87.7)	634 806 (87.0)	1 908 379 (88.0)	1 593 465 (88.9)	596 701 (87.6)	994 577 (89.5)
Census division						
New England	75 355 (2.6)	10 945 (1.5)	62 890 (2.9)	39 433 (2.2)	8855 (1.3)	28 893 (2.6)
Middle Atlantic	414 453 (14.3)	76 615 (10.5)	333 966 (15.4)	241 977 (13.5)	72 885 (10.7)	162 244 (14.6)
East North Central	504 300 (17.4)	129 150 (17.7)	377 339 (17.4)	290 373 (16.2)	115 798 (17.0)	181 135 (16.3)
West North Central	162 303 (5.6)	59 103 (8.1)	101 925 (4.7)	103 961 (5.8)	52 450 (7.7)	52 229 (4.7)
South Atlantic	608 638 (21.0)	179 497 (24.6)	431 554 (19.9)	406 880 (22.7)	173 016 (25.4)	235 587 (21.2)
East South Central	168 100 (5.8)	34 294 (4.7)	132 285 (6.1)	102 168 (5.7)	36 102 (5.3)	67 787 (6.1)
West South Central	420 250 (14.5)	141 554 (19.4)	279 751 (12.9)	286 788 (16.0)	128 059 (18.8)	162 244 (14.6)
Mountain	173 897 (6.0)	40 131 (5.5)	132 285 (6.1)	103 961 (5.8)	35 421 (5.2)	68 898 (6.2)
Pacific	373 877 (12.9)	59 103 (8.1)	314 449 (14.5)	215 091 (12.0)	58 580 (8.6)	153 354 (13.8)
Coverage type						
Parental	1 394 070 (48.1)	129 150 (17.7)	921 661 (42.5)	0	0	0
Policyholder	1 504 205 (51.9)	600 512 (82.3)	1 246 952 (57.5)	1 792 424 (100.0)	681 165 (100.0)	1 111 259 (100.0)
Total comorbidities^b^						
0	2 240 367 (77.3)	572 785 (78.5)	1 667 663 (76.9)	1 306 677 (72.9)	499 975 (73.4)	806 774 (72.6)
1	585 452 (20.2)	142 284 (19.5)	444 566 (20.5)	428 389 (23.9)	162 798 (23.9)	267 813 (24.1)
≥2	72 457 (2.5)	14 593 (2.0)	58 553 (2.7)	55 565 (3.1)	18 391 (2.7)	36 672 (3.3)
Depression	223 167 (7.7)	50 347 (6.9)	173 489 (8.0)	159 526 (8.9)	59 261 (8.7)	100 013 (9.0)
Annual deductible						
High, ≥$1000	423 148 (14.6)	92 667 (12.7)	329 629 (15.2)	179 242 (10.0)	57 899 (8.5)	115 571 (10.4)
Low, <$1000	2 475 127 (85.4)	636 995 (87.3)	1 838 984 (84.8)	1 613 182 (90.0)	623 266 (91.5)	995 688 (89.6)
Plan Type						
PPO	1 895 472 (65.4)	499 089 (68.4)	1 422 610 (65.6)	1 183 000 (66.0)	454 337 (66.7)	715 651 (64.4)
Comprehensive	43 474 (1.5)	13 134 (1.8)	30 361 (1.4)	16 132 (0.9)	7493 (1.1)	7779 (0.7)
EPO	49 271 (1.7)	4378 (0.6)	45 541 (2.1)	34 056 (1.9)	4087 (0.6)	30 004 (2.7)
HMO	388 369 (13.4)	131 339 (18.0)	258 065 (11.9)	267 071 (14.9)	137 595 (20.2)	135 574 (12.2)
POS	208 676 (7.2)	62 751 (8.6)	145 297 (6.7)	136 224 (7.6)	60 624 (8.9)	74 454 (6.7)
CDHP or HDHP	295 624 (10.2)	18 242 (2.5)	275 414 (12.7)	148 771 (8.3)	17 029 (2.5)	126 684 (11.4)

Abbreviations: CDHP, consumer-directed health plan; DCE, Dependent Coverage Expansion; EPO, exclusive provider organization; HDHP, high-deductible health plan; HMO, health maintenance organization; MSA, metropolitan statistical area; POS, point of service; PPO, preferred provider organization.

^a^ The treatment group included female young adults aged 23 to 25 years who were eligible for coverage, and the comparison group included female young adults aged 27 to 29 years who were not eligible for coverage. Patient characteristics are based on first year of enrollment. Data are presented as number (percentage) of enrollees unless otherwise indicated.

^b^ The Elixhauser Comorbidity Index classification system measures 30 comorbidity groups. Enrollees were classified into 3 categories based on the total number of comorbidities.

The proportion of enrollees in the treatment group using insurance for contraception decreased from 37.7% (95% CI, 37.6%-37.8%) in 2007-2009 to 36.5% (95% CI, 36.4%-36.5%) in 2011-2016 and increased from 34.2% (95% CI, 34.1%-34.3%) in 2007-2009 to 36.2% (95% CI, 36.1%-36.3%) in 2011-2016 in the comparison group, corresponding to a 3.2% relative reduction from the preimplementation to postimplementation periods in the treatment group compared with 5.9% increase in the comparison group (Table 2). In the adjusted DID analysis, implementation of the ACA-DCE and subsequent parental coverage eligibility was associated with a −2.9 (95% CI, −3.4 to −2.4) percentage point reduction in contraceptive service use by the treatment group after controlling for secular trends in the comparison group. Papanicolaou testing in the treatment group decreased from 53.6% (95% CI, 53.5%-53.7%) in 2007-2009 to 40.3% (95% CI, 40.3%-40.4%) in 2011-2016 and in the comparison group (24.7% relative decrease) from 56.9% (95% CI, 56.8%-57.0%) in 2007-2009 to 48.7% (95% CI, 48.7% -48.8%) in 2011-2016 (14.3% relative decrease). There was a −3.4 (95% CI, −3.9 to −3.0) percentage point reduction in Papanicolaou testing in the treatment group associated with ACA-DCE implementation in the adjusted DID analysis.

**Table 2.** Insurance Use for Contraception, Papanicolaou Testing, Emergency Department, and Well Visits Before (2007-2009) and After (2011-2016) DCE Implementation^a^

Type of service	Probability of use, % (95% CI)					
	Treatment group (4 076 596 person-years)		Comparison group (3 191 776 person-years)		Difference-in-differences^b^	
	Pre-DCE	Post-DCE	Pre-DCE	Post-DCE	Unadjusted	Adjusted
Contraception	37.7 (37.6 to 37.8)	36.5 (36.4 to 36.5)	34.2 (34.1 to 34.3)	36.2 (36.1 to 36.3)	−3.1 (−3.6 to −2.6)	−2.9 (−3.4 to −2.4)
Papanicolaou test	53.6 (53.5 to 53.7)	40.3 (40.3 to 40.4)	56.9 (56.8 to 57.0)	48.7 (48.7 to 48.8)	−4.9 (−5.4 to −4.4)	−3.4 (−3.9 to −3.0)
ED visit	15.9 (15.8 to 16.0)	15.7 (15.7 to 15.8)	15.8 (15.7 to 15.9)	15.1 (15.0 to 15.1)	0.6 (0.4 to 0.8)	0.4 (0.2 to 0.7)
Well visit	42.2 (42.1 to 42.3)	40.5 (40.4 to 40.5)	49.8 (49.7 to 49.1)	47.2 (47.1 to 47.3)	0.7 (0.3 to 1.1)	1.7 (1.3 to 2.1)

Abbreviations: DCE, Dependent Coverage Expansion; ED, emergency department.

^a^ Analyses were limited to individuals with at least 12 months of continuous enrollment. This analysis included 4 690 699 unique enrollees, with 7 268 372 person-years. All outcomes were measured annually. Explanatory variables included age, plan type, comorbidity category, high or low deductible, and residence in a micropolitan or metropolitan statistical area. The treatment group included female young adults aged 23 to 25 years who were eligible for coverage, and the comparison group included female young adults aged 27 to 29 years who were not eligible for coverage.

^b^ The difference-in-differences statistics represent the absolute percentage point difference in the probability of insurance use for each service by the treatment group after implementation of the Patient Protection and Affordable Care Act DCE, controlling for secular trends in the comparison group.

In placebo outcome DID analyses, there was a 0.4 (95% CI, 0.2-0.7) percentage point relative increase in the number of ED visits and a 1.7 (95% CI. 1.3-2.1) percentage point increase in well visits associated with ACA-DCE implementation by enrollees in the treatment group vs the comparison group.

In the sensitivity analyses limited to postimplementation period policyholders, there were no statistically significant differences in SRH insurance use between the treatment and comparison groups from the preimplementation to postimplementation periods. In the postimplementation period parental coverage–only adjusted models, there was an increase in the magnitude of differences for SRH services (4.8 [95% CI, −5.6 to −4.0] percentage point reduction in contraceptive insurance use; 6.3 [95% CI, −7.3 to −5.3] percentage point reduction in Papanicolaou testing). Across all other sensitivity analyses, results were qualitatively similar to the main findings (eTables 2-6 in the Supplement).

## Discussion

In this national cross-sectional study, large shifts from self-coverage to parental coverage under the ACA-DCE were associated with a reduction in insurance use for Papanicolaou testing and contraception services among commercially insured female young adults aged 23 to 25 years. Implementation of the ACA-DCE was also associated with a smaller yet statistically significant relative increase in ED and well visits.

These results suggest that female young adults newly eligible for parental coverage were less likely to use SRH services after ACA-DCE implementation, as captured by claims data; this findings may be attributable to enrollees electing to pay out of pocket for these services or forgoing these services. Because Papanicolaou testing is recommend for all female young adults (sexual activity notwithstanding), it is possible that factors other than confidentiality concerns were associated with insurance use behavior. Alternatively, confidentiality concerns about this procedure may be a relic of previous clinical guidelines that included sexual activity criteria or to well-documented confusion about the purpose of Papanicolaou testing.^24,25^ It is also possible that the sensitivity of Papanicolaou testing associated with ACE-DCE eligibility is attributable to a spillover effect from other SRH services; clinicians routinely perform Papanicolaou tests during contraceptive visits, and higher use in the comparison group may be associated with a higher likelihood of any type of SRH visit as opposed to actively seeking out Papanicolaou testing. The steady decrease in both groups over the study period may in part be associated with changes in cervical cancer screening guidelines, which reduced the recommended frequency of Papanicolaou testing from annually to biannually and then to once every 3 years.^26^ Guideline recommendations and changes were the same for female young adults aged 21 to 29 years across the study period and consequently should not have differentially influenced outcomes in the treatment vs comparison groups.

Our finding that contraceptive insurance use decreased among young adult enrollees deviates from prior research that suggests the ACA is associated with improved access to and use of contraceptive care.^27,28,29,30^ Findings are, however, consistent with the literature on confidentiality and SRH service use among adolescents and young adults.^12,31^ The large secular increase in contraceptive insurance use observed in the comparison group was likely associated with implementation of the contraceptive mandate, which required insurers to cover contraception without cost-sharing.^28,32^ Both SRH services that we examined in this study are covered at reduced or no cost-sharing under the ACA, highlighting an overlooked consequence of confidentiality issues; if young people are not using insurance to pay for or receive care, they are not benefitting from other ACA provisions, including the contraceptive and preventive service mandates.

Of importance, the observed reduction in insurance use for SRH care may or may not correspond to a reduction in service use. Confidentiality concerns lead some young people to seek SRH services outside traditional health care settings, most commonly, publicly funded family planning clinics, which are known for providing confidential and free or low-cost care if patients are uninsured or choose not to use their insurance.^33^ A 2016 survey of individuals seeking contraceptive care at Title X–funded facilities found that 25% of respondents with private coverage did not plan to use their insurance to pay for care, and of these, 31% indicated confidentiality concerns as the reason.^34^ This research was conducted after implementation of ACA coverage expansions and corroborates findings from our study while highlighting how some individuals with commercial coverage rely on publicly subsidized SRH care. Future research should examine whether and to what extent Title X–funded service use changed among young adults after ACA-DCE implementation and how recent changes to Title X program eligibility influenced the capacity of these facilities to provide confidential care.

Our findings suggest potential unintended consequences of the ACA-DCE. Insurance billing procedures used by private insurers routinely violate confidentiality for those with dependent coverage. Some states, insurers, and providers have enacted policies to protect dependent confidentiality.^10^ For example, the 2018 Massachusetts PATCH ACT allows dependents to request that information about service use be kept confidential in communications with the policyholder.^35^ Moving forward, it will be important to understand whether or not young adults are aware of these policies, if they take advantage of them, and their influence on health service use and outcomes. Ultimately, insurance use behavior of young adults may be associated with privacy perceptions as opposed to insurer policies or privacy breaches.

### Limitations

This study has limitations. First, given the large sample size, it is possible that small differences are statistically significant but not meaningful. Results should therefore be carefully interpreted, especially for smaller effect sizes. However, even a 1–percentage point change at the individual level translates conservatively to tens of thousands of forgone services at the population level. Second, differences between enrollees with parental and policyholder coverage that cannot be captured with insurance claims data could bias results if these differences are associated with sexual behavior and subsequent need for SRH care, although to our knowledge, there is no published literature to support this theory.

Third, differences between the treatment and comparison groups may also lead to selection bias if policyholder coverage “crowds-out” parental coverage for those in the treatment group with privacy concerns. However, prior research has demonstrated the opposite—eligible young adults were more likely to forgo self-coverage and use parental coverage after ACA-DCE implementation.^36^

Fourth, because these data did not allow identification of individual-level insurance coverage transitions, outcome estimates were based on a compositional change in the treatment group after policy implementation and included enrollees with both parental and policyholder coverage. As a result, these findings likely underestimate the association between ACA-DCE implementation and insurance use among those who gained parental coverage under the provision.

Fifth, our findings may not be generalizable to privately insured female young adults in the US. Although the use of narrow age ranges strengthens the internal validity of this study, it did not allow estimation of the association between the ACA-DCE and insurance use among young adults aged 19 to 22 years. The provision may have differentially influenced insurance use by younger enrollees who are at higher risk for unintended pregnancies^14^ and who may be more sensitive to confidentiality concerns.

## Conclusions

In this cross-sectional study, implementation of the ACA-DCE was associated with a decrease in insurance use for contraception and Papanicolaou testing, potentially because of privacy concerns. These findings raise questions about the capacity of parental coverage expansions to improve access to essential services among young adults.

## Notes

Supplement. **eMethods.** **eFigure 1.** Study sample selection **eFigure 2.** Compositional change in the treatment group after dependent coverage expansion: proportion of female enrollees with parental coverage **eFigure 3.** Unadjusted trends in emergency department (ED) visits among enrollees in the treatment (age 23 – 25) and comparison groups (27 – 29), 2007 - 2016 **eFigure 4.** Unadjusted trends in well visits among enrollees in the treatment (age 23 – 25) and comparison groups (27 – 29), 2007 - 2016 **eTable 1.** Pre-period (2007–2009) difference-in-differences model **eTable 2.** Sensitivity analyses A: Use of SRH services before (2007 – 2009) and after (2011–2016) DCE implementation: excluding women in the treatment group with parental coverage in the post-period **eTable 3.** Sensitivity analyses A: Use of SRH services before (2007 – 2009) and after (2011–2016) DCE implementation: excluding women in the treatment group with policyholder coverage in the post-period **eTable 4.** Sensitivity analyses B: Use of SRH services before (2007 – 2009) and three years after (2011-2013) DCE implementation **eTable 5.** Sensitivity analyses C: Use of SRH services before (2007 – 2009) with each year after DCE implementation as the post-period **eTable 6.** Sensitivity analyses D: Use of SRH services before (2007 – 2009) excluding enrollees who did not use any services **eTable 7.** Codes used to identify pap testing **eTable 8.** Codes used to identify contraception **eTable 9.** Codes used to identify emergency department visits **eTable 10.** Codes used to identify well visits
