# Supplementary material for: Association of the National Dependent Coverage Expansion With Insurance Use for Sexual and Reproductive Health Services by Female Young Adults
Source: JAMA Netw Open. 2020 Dec 18;3(12):e2030214. doi: 10.1001/jamanetworkopen.2020.30214 (PMC7749438; doi:10.1001/jamanetworkopen.2020.30214)
Supplement: Supplement. — eMethods. eFigure 1. Study sample selection eFigure 2. Compositional change in the treatment group after dependent coverage expansion: proportion of female enrollees with parental coverage eFigure 3. Unadjusted trends in emergency department (ED) visits among enrollees in the treatment (age 23 – 25) and comparison groups (27 – 29), 2007 - 2016 eFigure 4. Unadjusted trends in well visits among enrollees in the treatment (age 23 – 25) and comparison groups (27 – 29), 2007 - 2016 eTable 1. Pre-period (2007–2009) difference-in-differences model eTable 2. Sensitivity analyses A: Use of SRH services before (2007 – 2009) and after (2011–2016) DCE implementation: excluding women in the treatment group with parental coverage in the post-period eTable 3. Sensitivity analyses A: Use of SRH services before (2007 – 2009) and after (2011–2016) DCE implementation: excluding women in the treatment group with policyholder coverage in the post-period eTable 4. Sensitivity analyses B: Use of SRH services before (2007 – 2009) and three years after (2011-2013) DCE implementation eTable 5. Sensitivity analyses C: Use of SRH services before (2007 – 2009) with each year after DCE implementation as the post-period eTable 6. Sensitivity analyses D: Use of SRH services before (2007 – 2009) excluding enrollees who did not use any services eTable 7. Codes used to identify pap testing eTable 8. Codes used to identify contraception eTable 9. Codes used to identify emergency department visits eTable 10. Codes used to identify well visits [file jamanetwopen-e2030214-s001.pdf]

Supplemental Online Content

Ellison JE, Hanchate AD, Kazis LE, Cole MB. Association of the national Dependent Coverage Expansion with insurance use for sexual and reproductive health services by female young adults. *JAMA Netw Open.* 2020;3(12):e2030214. doi:10.1001/jamanetworkopen.2020.30214

eMethods.

eFigure 1. Study sample selection

eFigure 2. Compositional change in the treatment group after dependent coverage expansion: proportion of female enrollees with parental coverage

eFigure 3. Unadjusted trends in emergency department (ED) visits among enrollees in the treatment (age 23 – 25) and comparison groups (27 – 29), 2007 - 2016

eFigure 4. Unadjusted trends in well visits among enrollees in the treatment (age 23 – 25) and comparison groups (27 – 29), 2007 - 2016

eTable 1. Pre-period (2007–2009) difference-in-differences model

eTable 2. Sensitivity analyses A: Use of SRH services before (2007 – 2009) and after (2011–2016) DCE implementation: excluding women in the treatment group with parental coverage in the post-period

eTable 3. Sensitivity analyses A: Use of SRH services before (2007 – 2009) and after (2011–2016) DCE implementation: excluding women in the treatment group with policyholder coverage in the post-period

eTable 4. Sensitivity analyses B: Use of SRH services before (2007 – 2009) and three years after (2011-2013) DCE implementation

eTable 5. Sensitivity analyses C: Use of SRH services before (2007 – 2009) with each year after DCE implementation as the post-period

eTable 6. Sensitivity analyses D: Use of SRH services before (2007 – 2009) excluding enrollees who did not use any services

eTable 7. Codes used to identify pap testing

eTable 8. Codes used to identify contraception

eTable 9. Codes used to identify emergency department visits

eTable 10. Codes used to identify well visits

This supplemental material has been provided by the authors to give readers additional information about their work.

© 2020 Ellison JE et al. *JAMA Network Open.*

eMethods: Data Structure

The unit of analysis was person-year. Person-year was used instead of person-level, because individuals are enrolled in the data for different periods of time and consequently have a different likelihood of service use. Any enrollees with less than one person-year (12 months) of enrollment were excluded from the analysis.

Each row of our analytic data represents one person-year. If an individual was enrolled in a plan captured in the MarketScan data from 2011-2013, they are represented in the analytic data three times. Use of each services was dichotomized (yes/no) to indicate any use during the calendar year. Service use was treated as a binary outcome rather than a count for several reasons. First, each of the services of interest are provided over different time intervals. For example, pap testing is recommended once every three years, while the oral contraceptive is a daily medication filled several times in a year, and the IUD can last anywhere from 3-12 years. By dichotomizing the outcomes of interest at the person-year level, probability of service use is more standardized across enrollees and services. Additionally, the phenomenon of interest is the sensitivity of insurance use for confidential services by young adults eligible for parental coverage, which can be captured and presented most clearly by use or non-use.

eMethods: Statistical Model

For each outcome, we estimated the following least squares multivariable regression model:

$$(1)\ Y_{igst} = \beta_0 + \beta_1 T_g + \beta_2 P_t + \beta_3 (T_g * P_t) + X_i + \varphi_t + \sigma_s + \varepsilon_{igst}$$

where Y_{igst} indicates service use for individual *i*, of age *g*, living in state *s*, in year *t*. T_g is a dummy variable indicating whether age g falls in the treatment or comparison group and P_t is a dummy variable for whether period t is before or after DCE implementation (2011–2016). β_3 is the difference-in-difference coefficient, representing the average adjusted change in insurance use for each outcome in the post-implementation period attributable to parental coverage eligibility and controlling for secular trends in the comparison group. X_i is a vector of time-variant variables including age, plan type, residence in a micro- or metropolitan statistical area, and a categorical variable indicating the number of comorbidities (0, 1, 2, and ≥3) based on the Elixhauser comorbidity index. We also included fixed effects for state (σ_s) and year(φ_t), to account for unobserved time-invariant heterogeneity, and clustered standard errors at the state level.

© 2020 Ellison JE et al. *JAMA Network Open.*

eFigure 1. Study sample selection

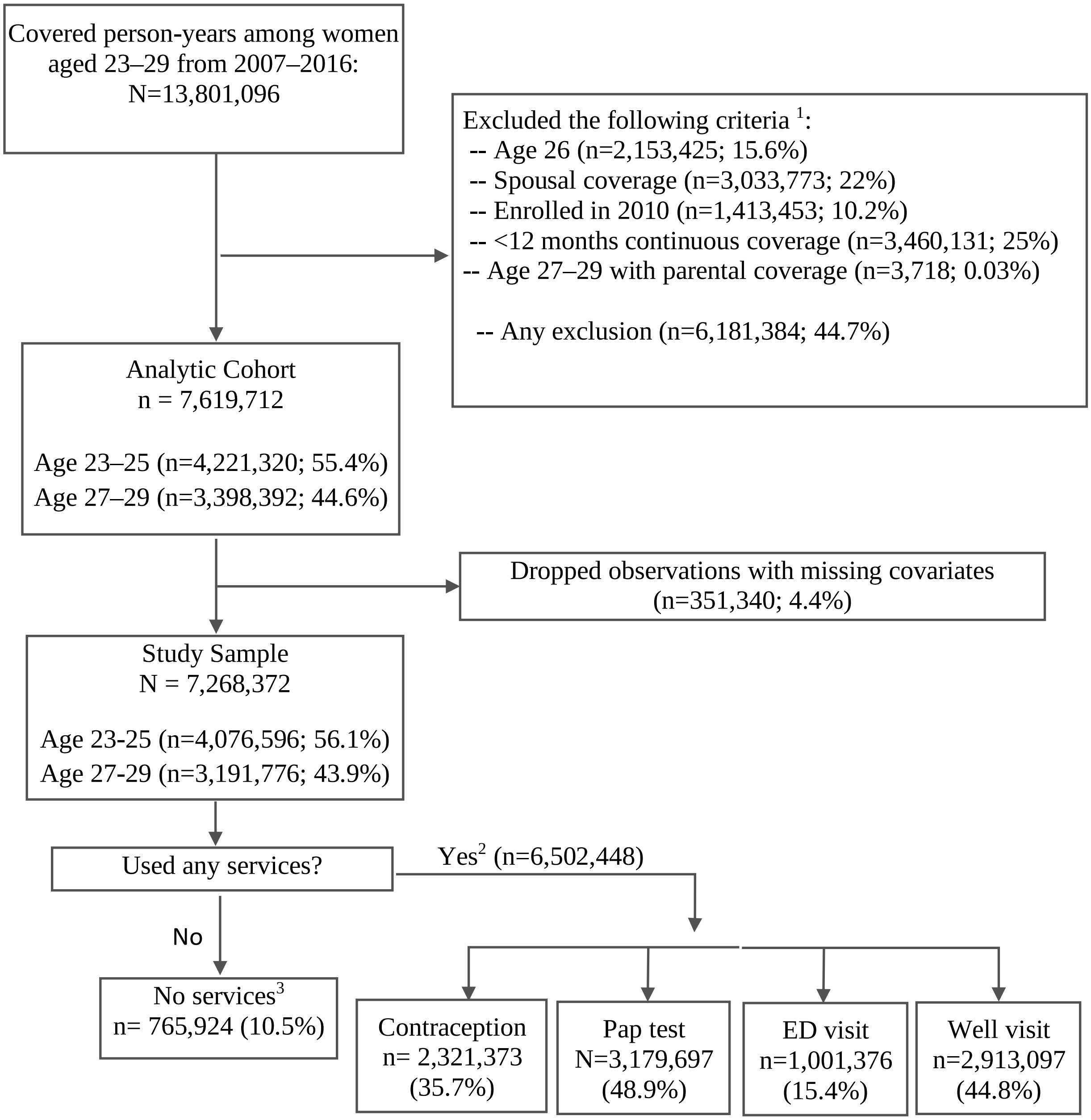

1. Exclusions are not mutually exclusive
2. Services are not mutually exclusive or limited to the four outcomes evaluated in this study
3. 'No services' refers to enrollees without claims for *any* health service during the study period

© 2020 Ellison JE et al. *JAMA Network Open.*

eFigure 2. Compositional change in the treatment group after dependent coverage expansion: proportion of female enrollees with parental coverage

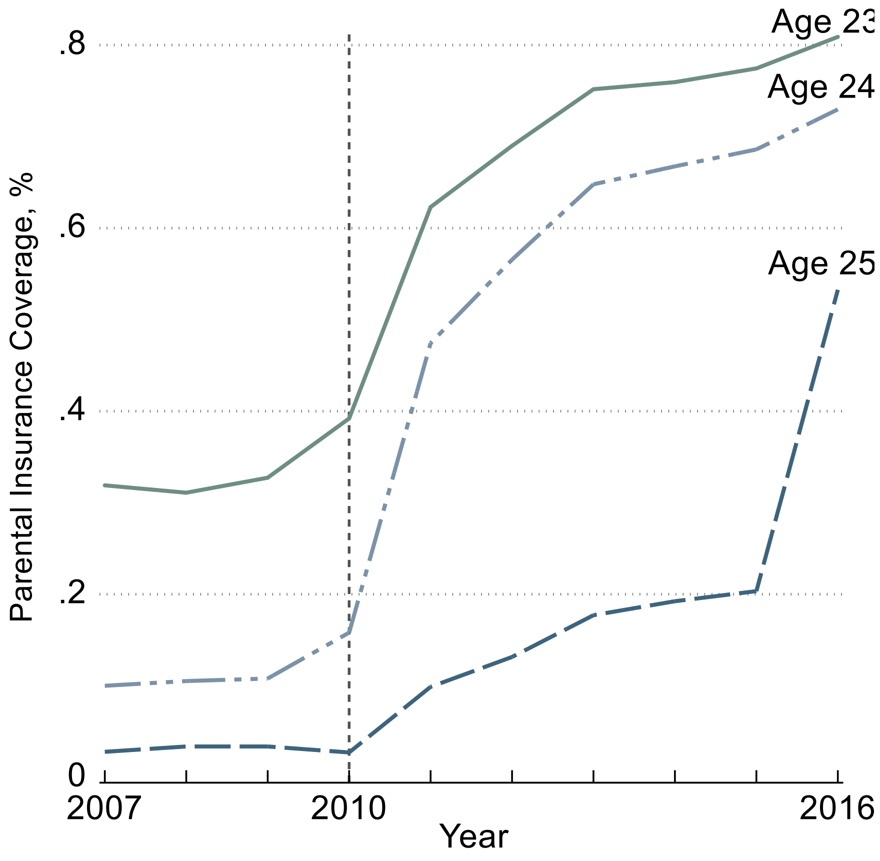

© 2020 Ellison JE et al. *JAMA Network Open.*

eFigure 3. Unadjusted trends in insurance use for emergency department (ED) visits among enrollees in the treatment (age 23 – 25) and comparison groups (27 – 29), 2007 - 2016

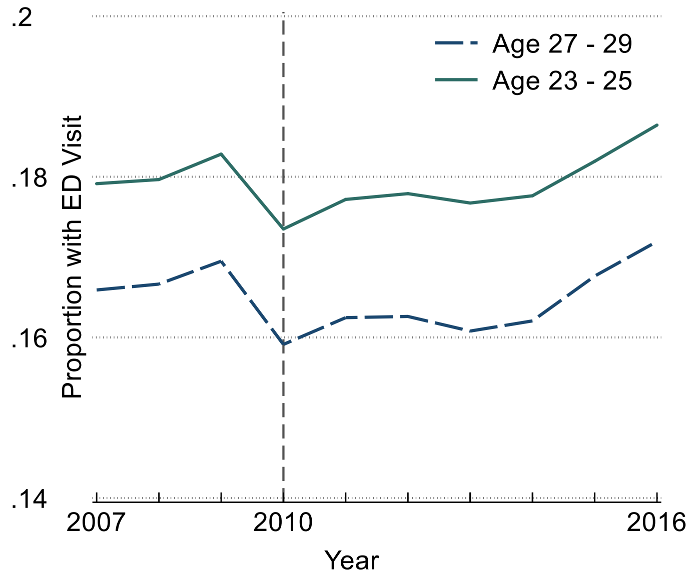

The denominator is person-years, and the numerator is any insurance use for an ED visit during the calendar year. The vertical line marks implementation of the national dependent coverage expansion, defining the pre- and post- periods. Unadjusted time trends demonstrate that enrollees in the treatment group was more likely to have an ED visit throughout the study period. In both groups, ED visits dropped slightly before ACA-DCE implementation and increased gradually in the post-period.

© 2020 Ellison JE et al. *JAMA Network Open.*

eFigure4. Unadjusted trends in insurance use for well visits among enrollees in the treatment (age 23 – 25) and comparison groups (27 – 29), 2007 - 2016

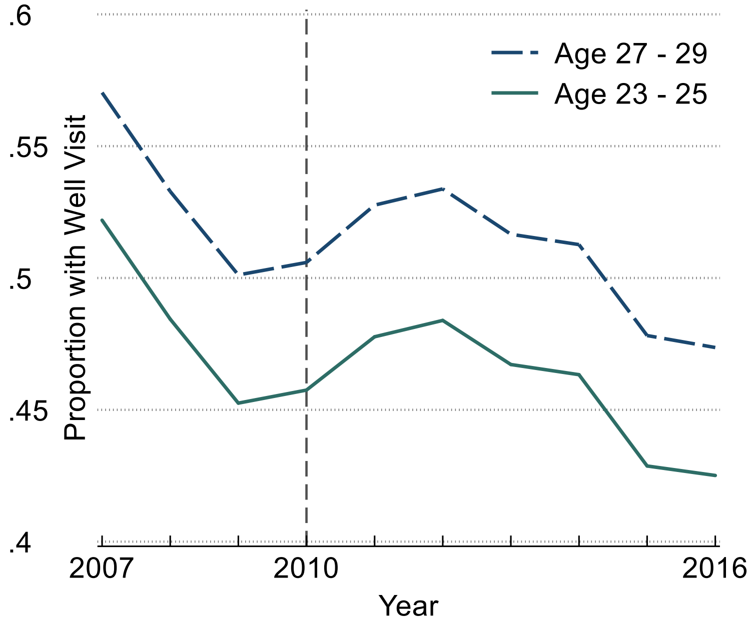

The denominator is person-years, and the numerator captures any insurance use for a well visit during the calendar year. The vertical line marks implementation of the national dependent coverage expansion, defining the pre- and post- periods. Unadjusted time trends demonstrate that enrollees in the treatment group were less likely to have a well visit throughout the study period. There was an brief uptick in both groups after ACA-DCE implementation, and the proportion of enrollees with an annual well visits began declining for both groups again in 2012.

© 2020 Ellison JE et al. *JAMA Network Open.*

eTable 1. Pre-period (2007–2009) difference-in-differences model

	DiD	95% CI	p-value
Contraception	-0.0021847	(-0.007 to 0.003)	0.401
Pap testing	0.0012387	(-0.0002 to 0.003)	0.109
ED visits	-0.0006472	(-0.002 to 0.001)	0.308
Well visits	-0.0050522	(-0.009 to -0.001)	0.021

As a formal test of the parallel trends assumption, we conducted the same DiD analysis as the primary model, using only data from the pre-DCE implementation years (January 2007 to December 2009). We split these three years into six bi-annual time periods. As with the primary analyses, we model each outcome as a function of the interaction between treatment status and a placebo policy implementation date (before or after time 3, or August 2008), adjusting for all covariates, state and year fixed effects, and standard errors clustered at the state-level. The interaction coefficient was statistically insignificant for contraception, pap testing, and ED visits, at $\alpha = 0.05$, and for well visits at $\alpha=0.01$ indicating similar pre-policy trends in service use between the treatment and comparison groups.

© 2020 Ellison JE et al. *JAMA Network Open.*

Sensitivity Analyses

A. Because we were able to identify coverage status, we repeated analyses with enrollees aged 23–25 who had only parental and only policyholder coverage in the post period, in order to identify the extent to which aggregate changes were due to coverage status. In the post-period policyholder only adjusted models, there were no statistically significant differences in contraception or pap test use between the treatment and comparison groups from the pre- to post-period. The difference in ED visits was significant, though the effect was small (DiD: 0.7%; 95% CI: 0.5 to 1.0) (**eTable 2**). In the post-period parental coverage only adjusted models, there was a notable increase in the magnitude of differences for contraceptive use, with a 4.8 (95% CI: -5.6 to -4.0) percentage point reduction, pap testing with a 6.3 (95% CI: -7.3 to -5.3) percentage point reduction, and well visits with a 2.4 (95% CI: 1.8 to 3.0) percentage point increase. There was a small, non-significant increase in ED use (**eTable 3**). These findings confirm that the aggregate change in insurance use for services among enrollees newly eligible for parental coverage can be attributed to the inclusion of women with parental coverage.

eTable 2. *Sensitivity analyses:* Use of SRH services before (2007 – 2009) and after (2011–2016) DCE implementation: excluding women in the treatment group with *parental coverage* in the post-period

	Contraception	Pap test	ED visit	Well visit
DiD	-0.6%	-0.02%	0.7%	1.0%
(95% CI)	(-1.1 to 1.5)	(-0.5 to 0.5)	(0.5 to 1.0)	(0.7 to 1.3)

eTable 3. *Sensitivity analyses:* Use of SRH services before (2007 – 2009) and after (2011–2016) DCE implementation: excluding women in the treatment group with *policyholder coverage* in the post-period

	Contraception	Pap test	ED visit	Well visit
DiD	-4.8%	-6.3%	0.3%	2.4%
(95% CI)	(-5.6 to -4.0)	(-7.3 to -5.3)	(-0.01 to 0.7)	(1.8 to 3.0)

© 2020 Ellison JE et al. *JAMA Network Open.*

B. We replicated the analyses excluding years 2014–2016, which may be subject to secondary effects of ACA coverage expansions (i.e. Medicaid and insurance exchanges) that could differentially impact the treatment and comparison groups. As in the primary analysis, there was a reduction in insurance use for all SRH services after excluding years 2014–2016, and a slight increase in ED visits. The magnitude of these reductions were similar to the primary analysis, suggesting that observed effects are largely a consequence of the DCE, as opposed to other changes in coverage options including ACA marketplace and Medicaid expansions, which have potential to influence the composition of the study population or subsequent insurance use behaviors (**eTable 4**).

eTable 4. *Sensitivity analyses:* Use of SRH services before (2007 – 2009) and *three years after (2011-2013)* DCE implementation

	Contraception	Pap test	ED visit	Well visit
DiD	-2.8%	-3.0%	0.3%	1.7%
(95% CI)	(-3.3 to -2.3)	(-3.4 to -2.7)	(0.1 to 0.6)	(1.3 to 2.1)

© 2020 Ellison JE et al. *JAMA Network Open.*

C. We estimated the change in each year post-DCE, relative to the pre-DCE baseline (**eTable 5**). This analysis helps to identify the extent to which insurance use for SRH care may be a function of other policy changes occurring during the post period (such as the contraceptive mandate, which went into effect in 2012). With a few exceptions, the magnitude of the DiD increased slightly for contraceptive use and pap testing with each post-period year. A similar pattern occurred with ED visits. The increase in the magnitude of the coefficient for each subsequent year corresponds to the increase in the proportion of enrollees in the treatment group with parental coverage (eFigure 2).

eTable 5. *Sensitivity analyses:* Use of SRH services before (2007 – 2009) with *each year after* DCE implementation as the post-period

	Contraception	Pap test	ED visit	Well visit
2011	-2.7%	-2.2%	0.2%	1.9%
(95% CI)	(-3.2 to -2.1)	(-2.6 to -1.8)	(0.004 to 0.5)	(-0.1 to 0.4)
2012	-3.0%	-3.6%	0.4%	1.8%
(95% CI)	(-3.5 to -2.4)	(-4.1 to -3.2)	(0.1 to 0.6)	(1.3 to 2.2)
2013	-3.1%	-3.3%	0.5%	1.6%
(95% CI)	(-3.9 to -2.3)	(-3.9 to -2.8)	(0.2 to 0.8)	(1.0 to 2.2)
2014	-3.4%	-3.4%	0.6%	2.1%
(95% CI)	(-4.1 to -2.7)	(-4.0 to -2.8)	(0.4 to 0.9)	(1.5 to 2.6)
2015	-2.8%	-3.8%	0.4%	1.1%
(95% CI)	(-3.6 to -2.1)	(-4.5 to -3.2)	(0.1 to 0.8)	(0.4 to 1.9)
2016	-3.4%	-4.6%	0.8%	2.0%
(95% CI)	(-4.3 to -2.6)	(-5.2 to -3.9)	(0.4 to 1.2)	(1.3 to 2.8)

© 2020 Ellison JE et al. *JAMA Network Open.*

D. Finally, we replicated analyses excluding enrollees who did not use any healthcare over the study period to address potential differences in care-seeking behavior between the treatment and comparison group more generally. After excluding enrollees who did not use any care (n=765,924), the magnitude of the effects increased very slightly across outcomes (**eTable 6**).

eTable 6. *Sensitivity analyses:* Use of SRH services before (2007 – 2009) and after (2011–2016) DCE implementation: excluding enrollees who did not use any services throughout the study period

	Contraception	Pap test	ED visit	Well visit
DiD	-3.0%	-3.9%	0.5%	1.8%
(95% CI)	(-3.5 to -2.4)	(-4.3 to -3.4)	(0.3 to 0.8)	(1.4 to 2.2)

© 2020 Ellison JE et al. *JAMA Network Open.*

**eTable 7.** Codes used to identify pap testing		
**Code**	**Definition**	**Code system**
88141	Cytopathology, cervical or vaginal (any reporting system), requiring interpretation by physician	CPT
88142	Cytopathology, cervical or vaginal (any reporting system), collected in preservative fluid, automated thin layer preparation; manual screening under physician supervision	CPT
88143	Cytopathology, cervical or vaginal (any reporting system), collected in preservative fluid, automated thin layer preparation; with manual screening and rescreening under physician supervision	CPT
88147	Cytopathology smears, cervical or vaginal; screening by automated system under physician supervision	CPT
88148	Cytopathology smears, cervical or vaginal; screening by automated system with manual rescreening under physician supervision	CPT
88150	Cytopathology, slides, cervical or vaginal; manual screening under physician supervision	CPT
88152	Cytopathology, slides, cervical or vaginal; with manual screening and rescreening under physician supervision	CPT
88153	Cytopathology, slides, cervical or vaginal; with manual screening and rescreening under physician supervision	CPT
88154	Cytopathology, slides, cervical or vaginal; with manual screening and computer-assisted re screening using cell selection and review under physician supervision	CPT
88164	Cytopathology, slides, cervical or vaginal (the Bethesda System); manual screening under physician supervision	CPT
88165	Cytopathology, slides, cervical or vaginal (the Bethesda System); with manual screening and rescreening under physician supervision	CPT
88166	Cytopathology, slides, cervical or vaginal (the Bethesda System); with manual screening and computer-assisted rescreening under physician supervision	CPT
88167	Cytopathology, slides, cervical or vaginal (the Bethesda System); with manual screening and computer-assisted rescreening using cell selection and review under physician supervision	CPT
88174	Cytopathology, cervical or vaginal (any reporting system), collected in preservative fluid, automated thin layer preparation; screening by automated system, under physician supervision	CPT
88175	Cytopathology, cervical or vaginal (any reporting system), collected in preservative fluid, automated thin layer	CPT

© 2020 Ellison JE et al. *JAMA Network Open.*

	preparation; with screening by automated system and manual rescreening or review, under physician supervision	
G0123	Screening cytopathology, cervical or vaginal (any reporting system), collected in preservative fluid, automated thin layer preparation, screening by cytotechnologist under physician supervision (G0123)	HCPCS
G0124	Screening cytopathology, cervical or vaginal (any reporting system), collected in preservative fluid, automated thin layer preparation, requiring interpretation by physician (G0124)	HCPCS
G0141	Screening cytopathology smears, cervical or vaginal, performed by automated system, with manual rescreening, requiring interpretation by physician (G0141)	HCPCS
G0143	Screening cytopathology, cervical or vaginal (any reporting system), collected in preservative fluid, automated thin layer preparation, with manual screening and rescreening by cytotechnologist under physician supervision (G0143)	HCPCS
G0144	Screening cytopathology, cervical or vaginal (any reporting system), collected in preservative fluid, automated thin layer preparation, with screening by automated system, under physician supervision (G0144)	HCPCS
G0145	Screening cytopathology, cervical or vaginal (any reporting system), collected in preservative fluid, automated thin layer preparation, with screening by automated system and manual rescreening under physician supervision (G0145)	HCPCS
G0147	Screening cytopathology smears, cervical or vaginal, performed by automated system under physician supervision (G0147)	HCPCS
G0148	Screening cytopathology smears, cervical or vaginal, performed by automated system with manual rescreening (G0148)	HCPCS
P3000	Screening papanicolaou smear, cervical or vaginal, up to three smears, by technician under physician supervision (P3000)	HCPCS
P3001	Screening papanicolaou smear, cervical or vaginal, up to three smears, requiring interpretation by physician (P3001)	HCPCS
Q0091	Screening papanicolaou smear; obtaining, preparing and conveyance of cervical or vaginal smear to laboratory (Q0091)	HCPCS
79500	Abnormal glandular Papanicolaou smear of cervix	ICD-9
79501	Papanicolaou smear of cervix with atypical squamous cells of undetermined significance (ASC-US)	ICD-9
79502	Papanicolaou smear of cervix with atypical squamous cells cannot exclude high grade squamous intraepithelial lesion (ASC-H)	ICD-9
79503	Papanicolaou smear of cervix with low grade squamous intraepithelial lesion (LGSIL)	ICD-9

© 2020 Ellison JE et al. *JAMA Network Open.*

79504	Papanicolaou smear of cervix with high grade squamous intraepithelial lesion (HGSIL)	ICD-9
79505	Cervical high risk human papillomavirus (HPV) DNA test positive	ICD-9
79506	Papanicolaou smear of cervix with cytologic evidence of malignancy	ICD-9
79507	Satisfactory cervical smear but lacking transformation zone	ICD-9
79508	Unsatisfactory cervical cytology smear	ICD-9
79509	Other abnormal Papanicolaou smear of cervix and cervical HPV	ICD-9
79510	Abnormal glandular Papanicolaou smear of vagina	ICD-9
79511	Papanicolaou smear of vagina with atypical squamous cells of undetermined significance (ASC-US)	ICD-9
79512	Papanicolaou smear of vagina with atypical squamous cells cannot exclude high grade squamous intraepithelial lesion (ASC-H)	ICD-9
79513	Papanicolaou smear of vagina with low grade squamous intraepithelial lesion (LGSIL)	ICD-9
79514	Papanicolaou smear of vagina with high grade squamous intraepithelial lesion (HGSIL)	ICD-9
79515	Vaginal high risk human papillomavirus (HPV) DNA test positive	ICD-9
79516	Papanicolaou smear of vagina with cytologic evidence of malignancy	ICD-9
79518	Unsatisfactory vaginal cytology smear	ICD-9
79519	Other abnormal Papanicolaou smear of vagina and vaginal HPV	ICD-9
79670	Abnormal glandular Papanicolaou smear of anus	ICD-9
79671	Papanicolaou smear of anus with atypical squamous cells of undetermined significance (ASC-US)	ICD-9
79672	Papanicolaou smear of anus with atypical squamous cells cannot exclude high grade squamous intraepithelial lesion (ASC-H)	ICD-9
79673	Papanicolaou smear of anus with low grade squamous intraepithelial lesion (LGSIL)	ICD-9
79674	Papanicolaou smear of anus with high grade squamous intraepithelial lesion (HGSIL)	ICD-9
79675	Anal high risk human papillomavirus (HPV) DNA test positive	ICD-9
79676	Papanicolaou smear of anus with cytologic evidence of malignancy	ICD-9
79677	Satisfactory anal smear but lacking transformation zone	ICD-9
79678	Unsatisfactory anal cytology smear	ICD-9
79679	Other abnormal Papanicolaou smear of anus and anal HPV	ICD-9

© 2020 Ellison JE et al. *JAMA Network Open.*

V7232	Encounter for Papanicolaou cervical smear to confirm findings of recent normal smear following initial abnormal smear	ICD-9
R85610	Atypical squamous cells of undetermined significance on cytologic smear of anus (ASC-US)	ICD-10
R85611	Atypical squamous cells cannot exclude high grade squamous intraepithelial lesion on cytologic smear of anus (ASC-H)	ICD-10
R85612	Low grade squamous intraepithelial lesion on cytologic smear of anus (LGSIL)	ICD-10
R85613	High grade squamous intraepithelial lesion on cytologic smear of anus (HGSIL)	ICD-10
R85619	Unspecified abnormal cytological findings in specimens from anus	ICD-10
R87610	Atypical squamous cells of undetermined significance on cytologic smear of cervix (ASC-US)	ICD-10
R87611	Atypical squamous cells cannot exclude high grade squamous intraepithelial lesion on cytologic smear of cervix (ASC-H)	ICD-10
R87612	Low grade squamous intraepithelial lesion on cytologic smear of cervix (LGSIL)	ICD-10
R87613	High grade squamous intraepithelial lesion on cytologic smear of cervix (HGSIL)	ICD-10
R87614	Cytologic evidence of malignancy on smear of cervix	ICD-10
R87615	Unsatisfactory cytologic smear of cervix	ICD-10
R87616	Satisfactory cervical smear but lacking transformation zone	ICD-10
R87619	Unspecified abnormal cytological findings in specimens from cervix uteri	ICD-10
R87620	Atypical squamous cells of undetermined significance on cytologic smear of vagina (ASC-US)	ICD-10
R87621	Atypical squamous cells cannot exclude high grade squamous intraepithelial lesion on cytologic smear of vagina (ASC-H)	ICD-10
R87622	Low grade squamous intraepithelial lesion on cytologic smear of vagina (LGSIL)	ICD-10
R87623	High grade squamous intraepithelial lesion on cytologic smear of vagina (HGSIL)	ICD-10
R87624	Cytologic evidence of malignancy on smear of vagina	ICD-10
R87625	Unsatisfactory cytologic smear of vagina	ICD-10
R87628	Other abnormal cytological findings on specimens from vagina	ICD-10
R87628	Other abnormal cytological findings on specimens from vagina	ICD-10
R87810	Cervical high risk human papillomavirus (HPV) DNA test positive	ICD-10
R87811	Vaginal high risk human papillomavirus (HPV) DNA test positive	ICD-10

© 2020 Ellison JE et al. *JAMA Network Open.*

R87820	Cervical low risk human papillomavirus (HPV) DNA test positive	ICD-10
Z124	Encounter for screening for malignant neoplasm of cervix	ICD-10

© 2020 Ellison JE et al. *JAMA Network Open.*

**eTable 8.** Codes used to identify contraception		
**Code**	**Definition**	**Code system**
11976	Removal, implantable contraceptive capsules	CPT
57170	Diaphragm or cervical cap fitting with instructions	CPT
58300	Insertion of intrauterine device (IUD)	CPT
58301	Removal of intrauterine device (IUD)	CPT
11981	Insertion, non-biodegradable drug delivery implant	HCPCS
11982	Removal, non-biodegradable drug delivery implant	HCPCS
11983	Removal with reinsertion, non-biodegradable drug delivery implant	HCPCS
J7300	Intrauterine copper contraceptive	HCPCS
J7301	Levonorgestrel-releasing intrauterine contraceptive system, 13.5 mg	HCPCS
J7302	Levonorgestrel-releasing intrauterine contraceptive system, 52 mg	HCPCS
S4989	Contraceptive intrauterine device (e.g., progestacert iud), including implants and supplies	HCPCS
Q0090	Levonorgestrel-releasing intrauterine contraceptive system, 13.5 mg	HCPCS
S4981	Insertion of levonorgestrel-releasing intrauterine system	HCPCS
J7297	Levonorgestrel-releasing intrauterine contraceptive system, 52 mg, 3 year duration	HCPCS
J7298	Levonorgestrel-releasing intrauterine contraceptive system, 52 mg	HCPCS
J7306	Levonorgestrel (contraceptive) implant system, including implants and supplies	HCPCS
J7307	Etonogestrel (contraceptive) implant system, including implant and supplies	HCPCS
J1050	Injection, medroxyprogesterone acetate, 1 mg	HCPCS
J7304	Contraceptive supply, hormone containing patch, each	HCPCS
J7303	Contraceptive supply, hormone containing vaginal ring, each	HCPCS
A4266	Diaphragm for contraceptive use	HCPCS
A4261	Cervical cap for contraceptive use	HCPCS
S4993	Contraceptive pills for birth control	HCPCS
J7298	Levonorgestrel-releasing intrauterine contraceptive system, 52 mg, 5 year duration	HCPCS
Z30011	Encounter for initial prescription *of* contraceptive *pills*	ICD-10
Z30012	Encounter for prescription *of emergency* contraception	ICD-10
Z30013	Encounter for initial prescription *of injectable* contraceptive	ICD-10
Z30014	Encounter for initial prescription of intrauterine contraceptive device	ICD-10
Z30018	Encounter for initial prescription *of other* contraceptives	ICD-10

© 2020 Ellison JE et al. *JAMA Network Open.*

Z30019	Encounter for initial prescription *of* contraceptives, *unspecified*	ICD-10
Z3009	Encounter for other general counseling and advice on contraception	ICD-10
Z3040	Encounter for surveillance of contraceptives, unspecified	ICD-10
Z3041	Encounter for surveillance of contraceptive pills	ICD-10
Z3042	Encounter for surveillance of injectable contraceptive	ICD-10
Z30430	Encounter for insertion of intrauterine contraceptive device	ICD-10
Z30431	Encounter for routine checking of intrauterine contraceptive device	ICD-10
Z30432	Encounter for removal of intrauterine contraceptive device	ICD-10
Z30433	Encounter for removal and reinsertion of intrauterine contraceptive device	ICD-10
Z3049	Encounter for surveillance of other contraceptives	ICD-10
Z308	Encounter for other contraceptive management	ICD-10
Z309	Encounter for contraceptive management, unspecified	ICD-10
99632	Intrauterine device malfunction	ICD-9
V2511	Encounter for insertion of intrauterine contraceptive device	ICD-9
V2501	General counseling on prescription of oral contraceptives	ICD-9
V2502	General counseling on initiation of other contraceptive measures	ICD-9
V2503	Encounter for emergency contraceptive counseling and prescription	ICD-9
V2509	Other general counseling and advice on contraceptive management	ICD-9
V2512	Encounter for removal of intrauterine device	ICD-9
V2513	Encounter for removal and reinsertion of intrauterine contraceptive device	ICD-9
V2540	Contraceptive surveillance, unspecified	ICD-9
V2541	Surveillance of contraceptive pill	ICD-9
V2543	Surveillance of implantable subdermal contraceptive	ICD-9
V2549	Surveillance of other contraceptive method	ICD-9
V2542	Surveillance of intrauterine contraceptive device	ICD-9
V2543	Surveillance of implantable subdermal contraceptive	ICD-9
V2549	Surveillance of other contraceptive method	ICD-9
V255	Insertion of implantable subdermal contraceptive	ICD-9
V258	Other specified contraceptive management	ICD-9
V259	Unspecified contraceptive management	ICD-9
V251	Encounter for insertion or removal of intrauterine contraceptive device	ICD-9
V254	Surveillance of previously prescribe contraceptive methods	ICD-9
*For ease of presentation, NDC codes are not included. There were 4 NDC codes used to identify the subdermal implant, 32 for the injectable, 14 for the intrauterine device, 9 for the patch, 8 for the ring, 63 for the diaphragm, and 547 for the oral contraceptive pill		

© 2020 Ellison JE et al. *JAMA Network Open.*

**eTable 9.** Codes used to identify emergency department visits		
**Code**	**Definition**	**Code system**
99281	Emergency department visit for the evaluation and management of a patient	CPT
99282		CPT
99283		CPT
99284		CPT
99285		CPT

© 2020 Ellison JE et al. *JAMA Network Open.*

**eTable 10.** Codes used to identify well visits		
**Code**	**Definition**	**Code system**
V700	Routine general medical examination at a health care facility	ICD-9
V703	Other general medical examination	ICD-9
V706	Health examination in population surveys	ICD-9
V708	Other specified general medical examinations	ICD-9
V709	Unspecified general medical examination	ICD-9
Z0000	Encounter for general adult medical examination without abnormal findings	ICD-10
Z008	Encounter for other general examination	ICD-10
Z0001	Encounter for general adult medical examination without abnormal findings	ICD-10
G0438	Counseling, Screening, and Prevention Services	CPT
G0439	Counseling, Screening, and Prevention Services	CPT
99385	New Patient Preventive Medicine Services	CPT
99386	New Patient Preventive Medicine Services	CPT
99387	New Patient Preventive Medicine Services	CPT
99395	Established Patient Preventive Medicine Services	CPT
99396	Established Patient Preventive Medicine Services	CPT
99397	Established Patient Preventive Medicine Services	CPT
Any of these codes billed on the same day and by the same provider as a sexual/reproductive health service code were not considered a well visit		

© 2020 Ellison JE et al. *JAMA Network Open.*
